# Effectiveness and safety of afatinib, gefitinib, and erlotinib for treatment-naïve elderly patients with epidermal growth factor receptor-mutated advanced non-small-cell lung cancer: a multi-institute retrospective study

**DOI:** 10.18632/aging.205395

**Published:** 2024-01-08

**Authors:** Ling-Jen Hung, Ping-Chih Hsu, Cheng-Ta Yang, Chih-Hsi Scott Kuo, John Wen-Cheng Chang, Chen-Yang Huang, Ching-Fu Chang, Chiao-En Wu

**Affiliations:** 1 Division of Hematology-Oncology, Department of Internal Medicine, Linkou Chang Gung Memorial Hospital, College of Medicine, Chang Gung University, Taoyuan 333, Taiwan; 2 Division of Hematology-Oncology, Department of Internal Medicine, Taoyuan General Hospital, Ministry of Health and Welfare, Taoyuan 330, Taiwan; 3 Division of Thoracic Oncology, Department of Thoracic Medicine, Linkou Chang Gung Memorial Hospital, College of Medicine, Chang Gung University, Taoyuan 333, Taiwan

**Keywords:** elderly patients, epidermal growth factor receptor, tyrosine kinase inhibitor, non-small-cell lung cancer, real-world evidence

## Abstract

Background: In real-world practice, most patients with lung cancer are diagnosed when they are aged ≥65 years. However, clinical trials tend to lack data for the elderly population. Therefore, we aimed to describe the effectiveness and safety of afatinib, gefitinib, and erlotinib for elderly patients with epidermal growth factor receptor (*EGFR*)-mutated advanced non-small-cell lung cancer (NSCLC).

Methods: Treatment-naïve patients with *EGFR*-mutated advanced NSCLC were enrolled at many hospitals in Taiwan. Patient characteristics and the effectiveness and safety of afatinib, gefitinib, and erlotinib were compared.

Results: This study enrolled 1,343 treatment-naïve patients with *EGFR*-mutated advanced NSCLC, of whom 554 were aged <65 years, 383 were aged 65–74 years, 323 were aged 75–84 years, and 83 were aged ≥85 years. For elderly patients, afatinib was more effective, with a median progression-free survival (PFS) of 14.7 months and overall survival (OS) of 22.2 months, than gefitinib (9.9 months and 17.7 months, respectively) and erlotinib (10.8 months and 18.5 months, respectively; PFS: *p* = 0.003; OS: *p* = 0.026). However, grade ≥3 adverse events, including skin toxicities, paronychia, mucositis, and diarrhea, were more frequently experienced by patients receiving afatinib than those receiving gefitinib or erlotinib.

Conclusions: This large retrospective study provides real-world evidence of the effectiveness and safety of EGFR-TKIs for elderly patients with EGFR-mutated advanced NSCLC, a population that is often underrepresented in clinical trials and real-world evidence. Afatinib was more effective as a first-line treatment than gefitinib or erlotinib for elderly patients with *EGFR*-mutated advanced NSCLC.

## INTRODUCTION

Lung cancer is the second most commonly diagnosed cancer and the leading cause of cancer-related death worldwide, with an estimated 2.2 million new cases and 1.8 million deaths in 2020 [1]. Non-small-cell lung cancer (NSCLC) represents 80%–85% of all lung cancer cases and is often diagnosed at an advanced stage [2]. There is significant geographical variation in epidermal growth factor receptor (*EGFR*) mutations, which are much more common in Asian (40%–60%) than in Western (10%–15%) NSCLC populations [3]. Activating *EGFR* mutations (e.g., exon 19 deletions and L858R) are predictive of progression-free survival (PFS), overall survival (OS), and response to tyrosine kinase inhibitors (TKIs) [4], of which afatinib, gefitinib, and erlotinib have been approved to treat *EGFR*-mutated NSCLC [4–6].

Lung cancer disproportionately affects older adults, with 71.1% of newly diagnosed patients being ≥65 years old and 36.2% being ≥75 years old [7]. This population often experiences physiological problems and increased comorbidities, with approximately half of those aged >75 years having two or more complications. Multiple factors, including polypharmacy, decreased social support, and limited economic resources, can affect the tolerability and effectiveness of cancer treatment for elderly patients [8]. The median age of patients was 63 years in the afatinib group and gefitinib group in the LUX-Lung 7 study [9], 61.5 years in the afatinib group in the LUX-Lung 3 study, 57 years in the gefitinib group in the IPASS study, and 65 years in the erlotinib group in the EURTAC study [4–6]. In the LUX-Lung 7 study [9], the only randomized study comparing afatinib and first-generation EGFR-TKIs, the benefit of afatinib for the subgroup (aged ≥65 years) receiving this therapy was nonsignificant, with a hazard ratio (HR) of 0.85 (95% confidence interval [CI] = 0.59–1.22) for PFS. Therefore, comparisons of the effectiveness and safety of these EGFR-TKIs approved for patients aged ≥65 years are limited.

The available real-world evidence for EGFR-TKI treatment of elderly patients is also limited. Therefore, this study aimed to describe the effectiveness and safety of afatinib, gefitinib, and erlotinib for treatment-naïve elderly patients (aged ≥65 years) with *EGFR*-mutated advanced NSCLC.

## MATERIALS AND METHODS

### Patients and data collection

Patient data were obtained from the Cancer Registry System in the part of the Chang Gung Research Database [10, 11]. The selected patients were diagnosed with *EGFR*-mutated NSCLC and treated with first-line EGFR-TKI monotherapy (gefitinib, erlotinib, or afatinib) between May 2014, when Taiwan’s National Health Insurance began to reimburse afatinib, and January 2018. *EGFR* mutation status was retrospectively reviewed, and only patients with exon 19 deletions and exon 21 L858R mutations were included in the study.

The clinical data of patients who received EGFR-TKIs as first-line treatments were retrospectively reviewed. Their clinicopathological features, including age, sex, smoking history, Eastern Cooperative Oncology Group performance status (PS) score, tumor involvement, *EGFR* mutation (exon 19 deletion or L858R), dose adjustment (reduction/interruption), drug discontinuation, tumor response, adverse events (AEs), and subsequent treatment, were obtained. The last follow-up time point in this study was September 2021.

### Treatment and response evaluation

The patients were treated with EGFR-TKIs administered once daily until disease progression determined based on radiological studies or intolerable AEs as evaluated by clinicians. The dose and schedule of the EGFR-TKIs were adjusted by clinicians according to the patient’s clinical condition and treatment-related AEs. The tumor response was evaluated most frequently with computed tomography and sometimes with chest radiography and/or additional positron emission tomography. The tumor response was evaluated according to the Response Evaluation Criteria in Solid Tumors (version 1.1). The detailed definitions of tumor response, including complete response, partial response, stable disease, progressive disease, and not assessed, as well as definitions of PFS and OS, were described in our previous study [12].

### Adverse events

Data on AEs were collected from electronic medical records and graded according to the National Cancer Institute Common Terminology Criteria for Adverse Events (version 4.0). Dose adjustments (reductions or interruptions) and drug discontinuations or withdrawals due to AEs were recorded.

### Statistical analysis

Continuous variables were compared using the *t*-test or analysis of variance. Categorical variables were compared using the chi-square test or Fisher’s exact test. A series of univariate Cox proportional hazards models were applied to initially screen for potential factors associated with PFS and OS. Those variables with *p*-values <0.05 in the univariate Cox analysis were included in a multivariate Cox model. A two-sided *p*-value of <0.05 was considered statistically significant. All statistical analyses were performed using SPSS (2011 release; IBM Corp., Armonk, NY, USA), SPSS Statistics for Windows (version 20.0; IBM Corp.), and R statistical software (version 4.0.5) [13].

## RESULTS

### Comparison of patient characteristics between younger (aged <65 years) and older (aged ≥65 years) patients

This study included 1343 treatment-naïve patients with *EGFR*-mutated advanced NSCLC, of whom 554 were aged <65 years and 789 were aged ≥65 years. Compared to older patients, younger patients showed better PS, were less likely to be nonsmokers, were more likely to have stage IV disease, and had a higher incidence of brain, bone, and distant lymph node metastasis. Tumor morphology did not differ significantly between younger and older patients (*p* = 0.273). The exon 19 deletion was more common in older patients (56.4% vs. 44.6%), while the L858R point mutation was more common in younger patients (55.4% vs. 43.6%, *p* < 0.0001). Younger patients were more frequently treated with afatinib than older patients (58.3% vs. 39.6%, *p* < 0.0001). However, afatinib remained the TKI of choice for older patients compared to erlotinib or gefitinib. The characteristics of younger and older patients are presented in Supplementary Table 1.

Progression-free survival did not differ significantly between patients aged <65 years and those aged ≥65 years treated with any of the EGFR-TKIs considered (*p* = 0.568; gefitinib: *p* = 0.459; erlotinib: *p* = 0.920; afatinib: *p* = 0.858). However, patients aged <65 years had significantly longer OS than those aged ≥65 years when treated with one of the three EGFR-TKIs (median of 25.5 vs. 20.1 months, *p* < 0.0001; gefitinib: median of 22.5 vs. 17.7 months, *p* = 0.035; erlotinib: median of 23.7 vs. 18.5 months, *p* = 0.049; afatinib: median of 28.5 vs. 22.2 months, *p* = 0.018; Supplementary Figure 1).

### Characteristics of elderly patients (aged ≥65 years)

Elderly patients (aged ≥65 years) were further divided into three age groups and differences in their characteristics were examined. There were 383 patients aged 65–74 years, 323 aged 75–84 years, and 83 aged ≥85 years. The different age groups did not differ significantly in sex, smoking status, tumor morphology, and disease stage. PS worsened with age. Interestingly, the L858R point mutation was more common in patients aged ≥85 years (71.1%) than in those aged 75–84 (56.0%) or 65–74 (53.5%) years (*p* = 0.014). In addition, the choice of EGFR-TKI was age-dependent (*p* < 0.0001); gefitinib or erlotinib was prescribed more frequently as age increased, while afatinib was prescribed less frequently. The patients’ characteristics and the distribution of EGFR-TKIs among the age groups are summarized in Table 1.

**Table 1:** Patients’ characteristics of elderly patients (age ≥ 65 years).

**Characteristics**	**Age (years)**			***p*-Value**
	**65–74 (N=383)**	**75–84 (N=323)**	**≥85 (N=83)**	
Sex				0.307
Male	158 (41.3%)	115 (35.6%)	32 (38.6%)	
Female	225 (58.7%)	208 (64.4%)	51 (61.4%)	
Performance status				<0.0001
0	65 (17.0%)	36 (11.1%)	4 (4.8%)	
1	251 (65.5%)	193 (59.8%)	39 (47.1%)	
2	37 (9.7%)	55 (17.0%)	27 (32.5%)	
3	20 (5.2%)	21 (6.5%)	9 (10.8%)	
4	10 (2.6%)	18 (5.6%)	4 (4.8%)	
Smoking				0.270
No	290 (75.7%)	244 (75.6%)	69 (83.1%)	
Yes	80 (20.9%)	64 (19.8%)	14 (16.9%)	
Unknown	13 (3.4%)	15 (4.6%)	0	
Tumor morphology				0.418
Adenocarcinoma	376 (98.2%)	313 (96.9%)	82 (98.8%)	
Non-adenocarcinoma	7 (1.8%)	10 (3.1%)	1 (1.2%)	
Mutation				0.014
Exon 19 deletion	178 (46.5%)	142 (44.0%)	24 (28.9%)	
L858R	205 (53.5%)	181 (56.0%)	59 (71.1%)	
Stage				0.118
III	35 (9.1%)	21 (6.5%)	11 (13.3%)	
IV	348 (90.9%)	302 (93.5%)	72 (86.7%)	
EGFR-TKI				<0.0001
Afatinib	178 (46.5%)	114 (35.3%)	20 (24.1%)	
Erlotinib	133 (34.7%)	95 (29.4%)	31 (37.3%)	
Gefitinib	72 (18.8%)	114 (35.3%)	32 (38.6%)	
Liver metastasis				0.266
Yes	53 (13.8%)	33 (10.2%)	8 (9.6%)	
No	330 (86.2%)	290 (89.8%)	75 (90.4%)	
Brain metastasis				0.152
Yes	119 (31.1%)	92 (28.5%)	17 (20.5%)	
No	264 (68.9%)	231 (71.5%)	66 (79.5%)	
Lung metastasis				0.473
Yes	151 (39.4%)	138 (42.7%)	30 (36.1%)	
No	232 (60.6%)	185 (57.3%)	53 (63.9%)	
Bone metastasis				0.052
Yes	173 (45.2%)	125 (38.7%)	27 (32.5%)	
No	210 (54.8%)	198 (61.3%)	56 (67.5%)	
Pleura metastasis				0.034
Yes	163 (42.6%)	169 (52.3%)	40 (48.2%)	
No	220 (57.4%)	154 (47.7%)	43 (51.8%)	
Adrenal metastasis				0.154
Yes	39 (10.2%)	33 (10.2%)	3 (3.6%)	
No	344 (89.8%)	290 (89.8%)	80 (96.4%)	
Distant lymph node metastasis				0.520
Yes	38 (9.9%)	32 (9.9%)	5 (6.0%)	
No	345 (90.1%)	291 (90.1%)	78 (94.0%)	
Pericardia metastasis				0.347
Yes	4 (1.0%)	6 (1.9%)	0	
No	379 (99.0%)	317 (98.1%)	83 (100.0%)	
Peritoneal metastasis				0.777
Yes	2 (0.5%)	2 (0.6%)	0	
No	381 (99.5%)	321 (99.4%)	83 (100.0%)	

Footnote: Continuous variables were compared using a t-test or analysis of variance. Categorical variables were compared using a Chi-square or Fisher’s exact test.

Abbreviations: EGFR-TKI, epidermal growth factor receptor-tyrosine kinase inhibitor.

### Outcomes of EGFR-TKI therapy for elderly patients

Of the 789 elderly patients, 218 were treated with gefitinib, 259 with erlotinib, and 312 with afatinib. The effectiveness of the three EGFR-TKIs was evaluated in relation to objective response rate (ORR) and disease control rate (DCR). In the elderly patients, afatinib had a marginally higher ORR (68.6%) than gefitinib (58.7%) and erlotinib (62.2%; *p* = 0.054), but a significantly higher DCR (83.3%) than gefitinib (74.3%) and erlotinib (77.2%; *p* = 0.032). However, when the data were analyzed separately for each age subgroup, afatinib had numerically higher ORRs than gefitinib or erlotinib, although the differences were nonsignificant. Afatinib had a significantly higher DCR than gefitinib or erlotinib in patients aged 65–74 years (*p* = 0.027) but not in the other subgroups. The results are summarized in Table 2.

**Table 2:** The objective response rates (ORR) and disease control rates (DCR) of epidermal growth factor receptor-tyrosine kinase inhibitors (EGFR-TKIs) among elderly patients (age ≥ 65 years).

**Characteristics**	**EGFR-TKIs**			***p*-Value**
	**Gefitinib**	**Erlotinib**	**Afatinib**	
Age (years)				
Overall≥65	(N=218)	(N=259)	(N=312)	
ORR	128 (58.7%)	161 (62.2%)	214 (68.6%)	0.054
DCR	162 (74.3%)	200 (77.2%)	260 (83.3%)	0.032
65–75	(N=72)	(N=133)	(N=178)	
ORR	49 (68.1%)	84 (63.2%)	125 (70.2%)	0.417
DCR	56 (77.8%)	102 (76.7%)	156 (87.6%)	0.027
75–55	(N=114)	(N=95)	(N=114)	
ORR	66 (57.9%)	62 (65.3%)	76 (66.7%)	0.343
DCR	84 (73.7%)	74 (77.9%)	91 (79.8%)	0.531
≥85	(N=32)	(N=31)	(N=20)	
ORR	13 (40.6%)	15 (48.4%)	13 (65.0%)	0.229
DCR	22 (68.8%)	24 (77.4%)	13 (65.0%)	0.591

Footnote: The tumor response was evaluated most frequently by computed tomography and sometimes by chest radiography and/or additional positron emission tomography. The tumor response was evaluated according to the Response Evaluation Criteria in Solid Tumors (version 1.1).

**Figure 1:**
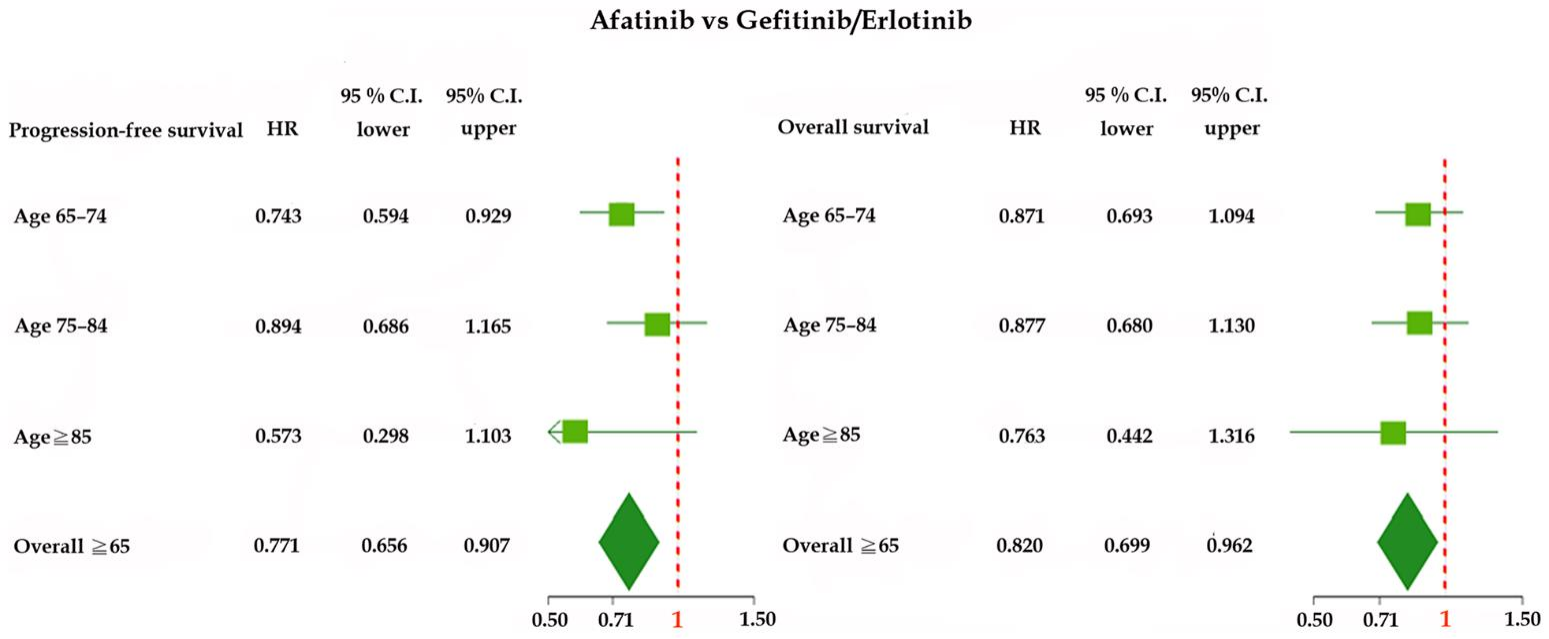
**Forest plot of progression-free survival (PFS) and overall survival (OS) of elderly patients (age ≥65 years) treated with afatinib, gefitinib, or erlotinib.** Abbreviations: HR, hazard ratio; CI, confidence interval.

**Figure 2:**
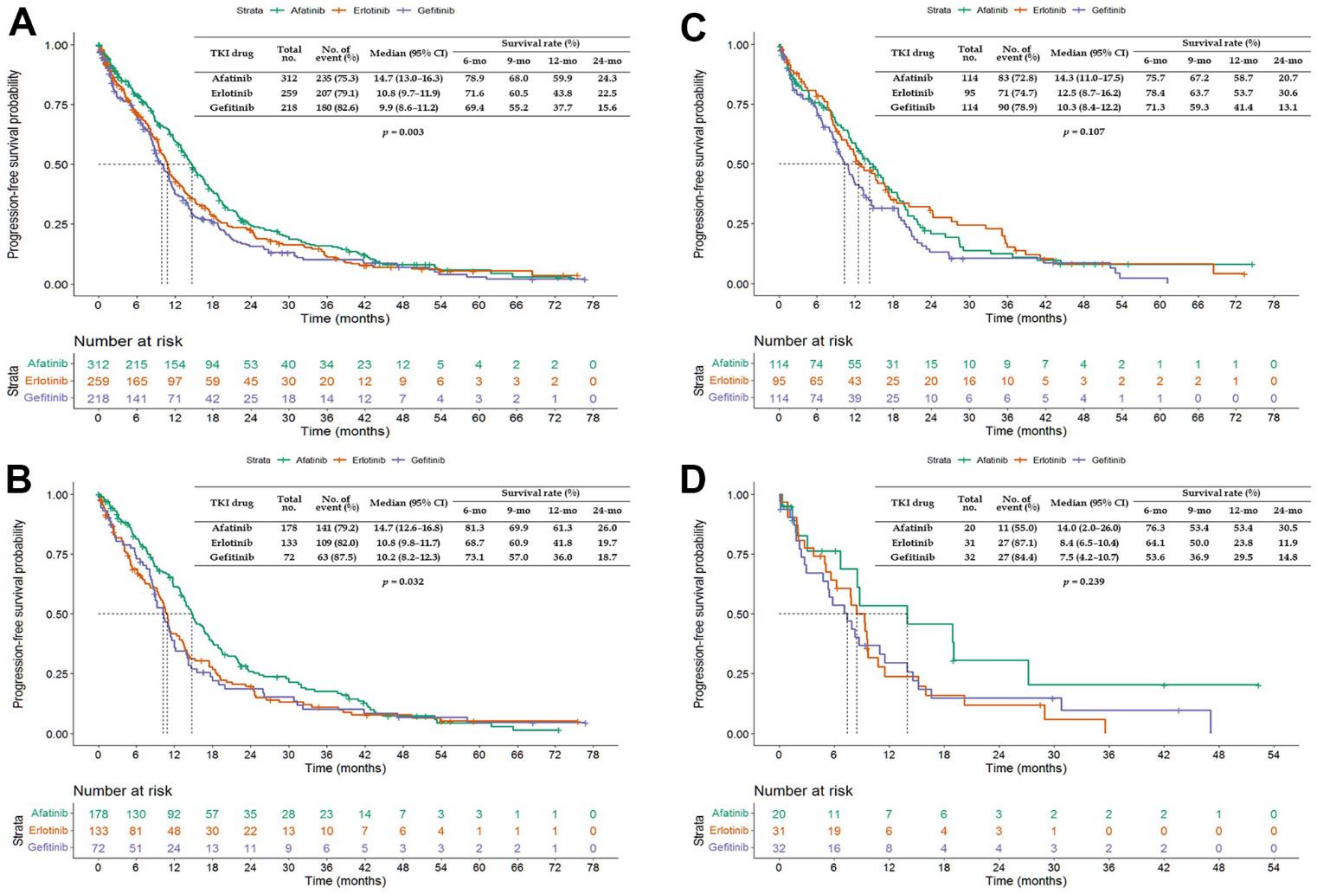
**Kaplan–Meier curves of progression-free survival (PFS) of elderly patients treated with afatinib, erlotinib, or gefitinib.** (**A**) Overall age ≥65 years; (**B**) age 65–74 years; (**C**) age 75–84 years; and (**D**) age ≥85 years. Abbreviations: TKI, tyrosine kinase inhibitor; CI, confidence interval.

**Figure 3:**
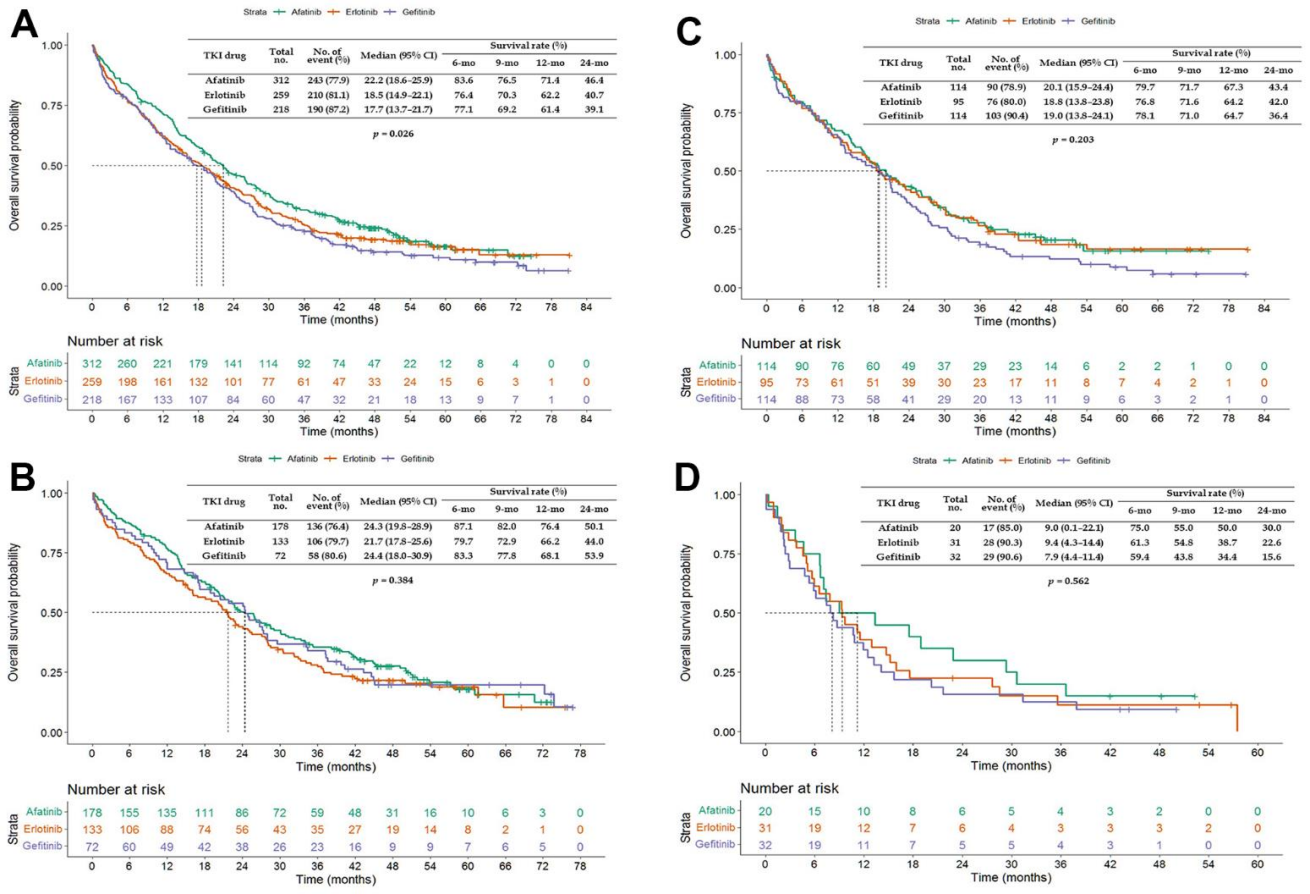
**Kaplan–Meier curves of overall survival (OS) of elderly patients treated with afatinib, erlotinib, or gefitinib.** (**A**) Overall age ≥65 years; (**B**) age 65–74 years; (**C**) age 75–84 years; and (**D**) age ≥85 years. Abbreviations: TKI, tyrosine kinase inhibitor; CI, confidence interval.

The PFS and OS of patients aged ≥65 years treated with afatinib were more favorable (PFS: HR = 0.771, 95% CI = 0.656–0.907; OS: HR = 0.820, 95% CI = 0.699–0.962) than those of patients treated with erlotinib or gefitinib (Figure 1). Patients aged ≥65 years treated with afatinib had significantly longer PFS (median of 14.7 vs. 9.9 and 10.8 months, *p* = 0.003; Figure 2A) and OS (median of 22.2 vs. 17.7 and 18.5 months, *p* = 0.026; Figure 3A) than those treated with gefitinib or erlotinib. However, when the data were analyzed separately for each age subgroup (Figures 1–3), only PFS of patients aged 65–74 years differed significantly (*p* = 0.032; Figure 2B).

**Figure 4:**
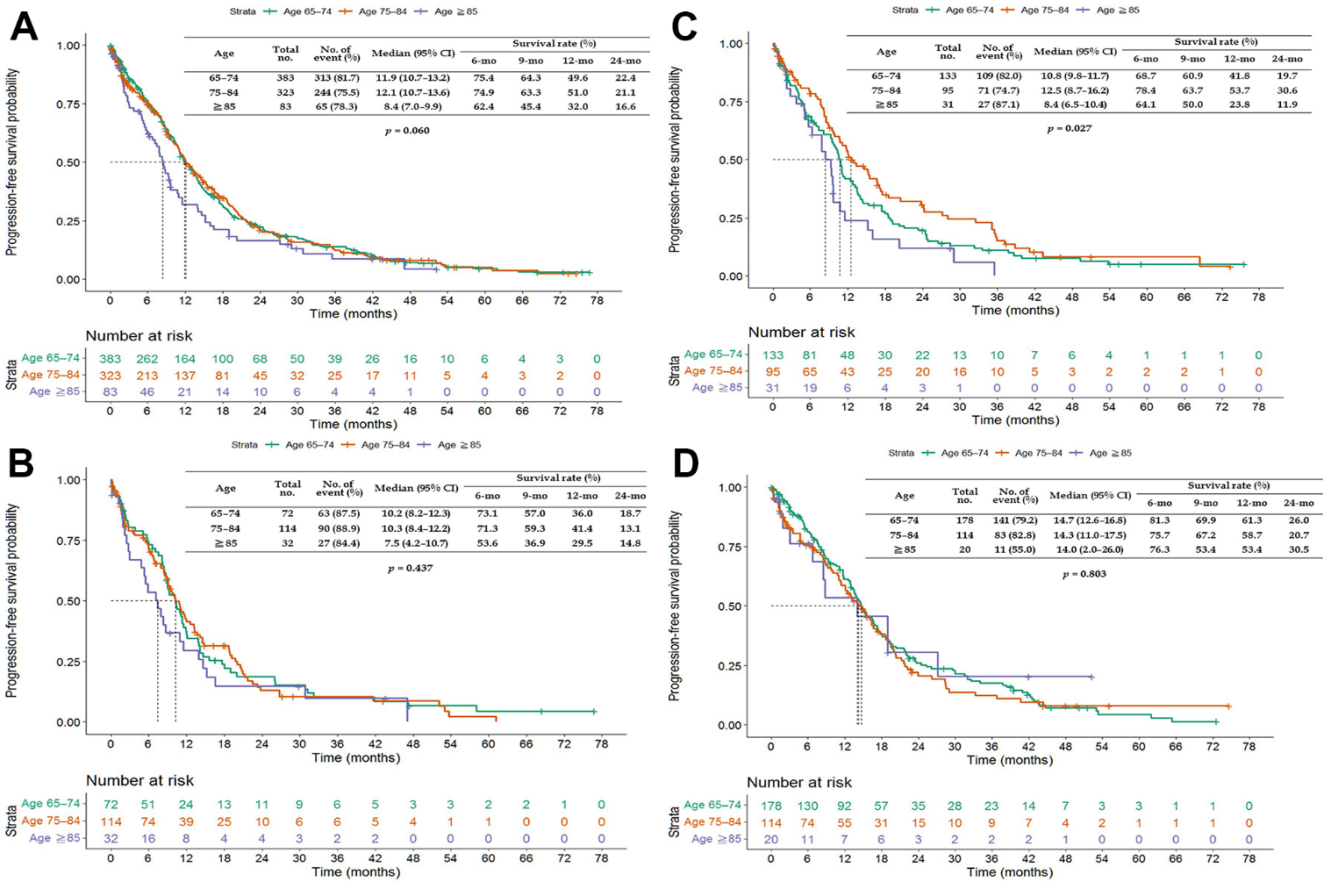
**Kaplan–Meier curves of progression-free survival (PFS) of elderly patients (age ≥65 years) treated with epidermal growth factor receptor-tyrosine kinase inhibitors (EGFR-TKIs).** (**A**) All EGFR-TKIs; (**B**) gefitinib; (**C**) erlotinib; and (**D**) afatinib. Abbreviation: CI, confidence interval.

**Figure 5:**
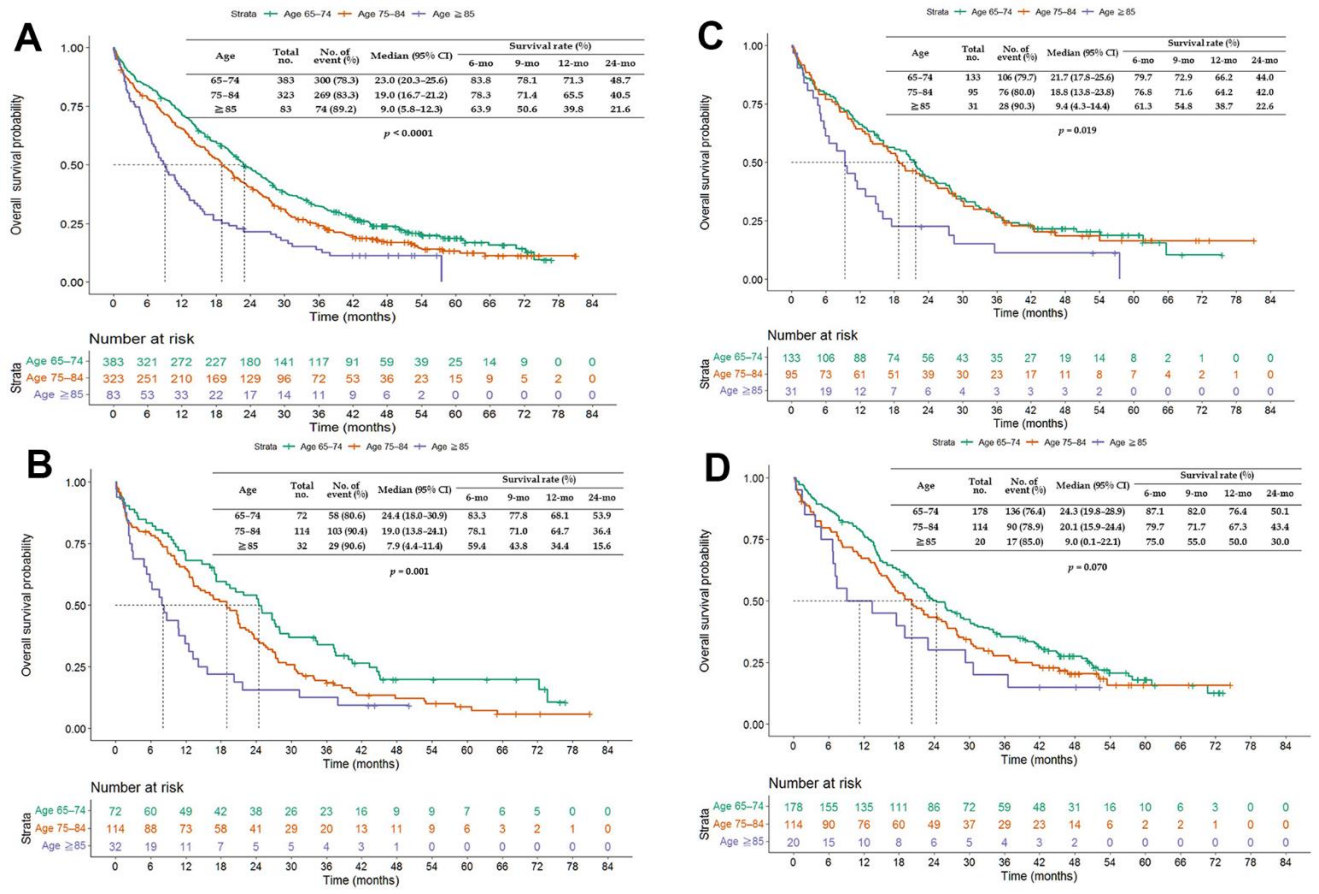
**Kaplan–Meier curves of overall survival (OS) of elderly patients (age ≥65 years) treated with epidermal growth factor receptor-tyrosine kinase inhibitors (EGFR-TKIs).** (**A**) All EGFR-TKIs; (**B**) gefitinib; (**C**) erlotinib; and (**D**) afatinib. Abbreviation: CI, confidence interval.

The PFS of patients aged 65–74 and 75–84 years was longer than that of patients aged ≥85 years when treated with any of the three EGFR-TKIs (*p* = 0.060) or erlotinib (*p* = 0.027) but not with gefitinib (*p* = 0.437) or afatinib (*p* = 0.803; Figure 4). The OS of patients aged 65–74 and 75–84 years was longer than that of patients aged ≥85 years when treated with any of the three EGFR-TKIs (*p* < 0.0001), gefitinib (*p* = 0.001), or erlotinib (*p* = 0.019; Figure 5).

### Adverse events of EGFR-TKIs

The AEs of EGFR-TKIs in elderly patients are presented in Table 3. The most common AEs of EGFR-TKIs, including skin toxicities, paronychia, mucositis, and diarrhea, were analyzed. The patients treated with afatinib experienced more AEs, as well as more grade ≥3 AEs, than those treated with gefitinib or erlotinib. In addition, more patients receiving afatinib required dose reductions or discontinuation compared to those treated with gefitinib or erlotinib.

**Table 3:** Adverse events of epidermal growth factor receptor-tyrosine kinase inhibitors (EGFR-TKIs) among elderly patients (age ≥ 65 years).

**Characteristics**	**EGFR-TKIs**			***p*-Value**
	**Gefitinib** **(N=218)**	**Erlotinib** **(N=259)**	**Afatinib** **(N=312)**	
Dose-reduction	25 (11.5%)	38 (14.7%)	122 (39.1%)	<0.0001
Dose discontinuation	9 (4.1%)	27 (10.4%)	46 (14.7%)	<0.001
Skin				
≥Grade 3	4 (1.8%)	10 (3.9%)	19 (6.1%)	0.052
Any grades	100 (45.9%)	154 (59.5%)	191 (61.2%)	0.001
Paronychia				
≥Grade 3	1 (0.5%)	6 (2.3%)	16 (5.1%)	0.006
Any grades	52 (23.9%)	67 (25.9%)	160 (51.3%)	<0.0001
Mucositis				
≥Grade 3	0	3 (1.1%)	6 (1.9%)	0.122
Any grades	27 (12.4%)	34 (13.1%)	112 (35.9%)	<0.0001
Diarrhea				
≥Grade 3	5 (2.3%)	4 (1.5%)	29 (9.3%)	<0.0001
Any grades	76 (34.9%)	90 (34.7%)	230 (73.7%)	<0.0001

Footnote: The EGFR-TKIs’ dose and schedule were adjusted by clinicians based on the patient’s clinical condition and treatment-related AEs. Data on AEs were collected from electronic medical records and graded according to National Cancer Institute Common Terminology Criteria for Adverse Events (version 4.0).

### Univariate and multivariate analysis of prognostic factors of progression-free survival of elderly patients

A univariate analysis was performed to explore the possible prognostic factors of PFS of elderly patients treated with EGFR-TKIs (Table 4). Patients with a PS score of 2–4, stage IV disease, and ≥4 metastatic sites had significantly worse PFS. Patients with liver, brain, bone, pleural, adrenal, and pericardial metastasis showed significantly worse PFS. Treatment with gefitinib also resulted in significantly worse PFS.

**Table 4:** Univariate and multivariate analysis of prognostic factors of progression-free survivals (PFS) for elderly patients (age ≥ 65 years).

**Parameters**	**No**	**Univariate analysis**				**Multivariate analysis**		
		**Median** **(months)**	**95% CI**	***p-*Value**		**Hazard ratio**	**95% CI**	***p-*Value**
Age (years)				0.060		-		
65–74	383	11.9	10.7–13.2					
75-84	323	12.1	10.7–13.6					
≥85	83	8.4	7.0–9.9					
Sex				0.474		-		
Male	305	11.0	9.3–12.8					
Female	484	11.8	10.6–13.0					
Performance status				<0.0001				
0–1	588	13.3	12.2–14.4			Reference		
2–4	201	6.8	5.1–8.5			1.73	1.43–2.09	<0.0001
Smoking				0.078		-		
Yes	158	9.7	7.4–12.0					
No	603	12.0	10.9–13.0					
Unknown	28	10.3	8.9–11.7					
Tumor morphology				0.102		-		
Adenocarcinoma	771	11.7	10.8–12.6					
Non-adenocarcinoma	18	4.6	0.1–9.4					
Mutation				0.646		-		
19del	344	12.9	11.5–14.2					
L858R	445	11.0	10.1–11.9					
Stage				<0.0001				
IIIB	67	27.2	18.6–35.7			Reference		
IV	722	11.1	10.2–11.9			1.75	1.26–2.45	<0.001
Lung metastasis				0.113		-		
Yes	319	11.1	10.1–12.2					
No	470	12.0	10.7–13.4					
Liver metastasis				<0.0001				
Yes	94	8.9	7.3–10.5			1.34	1.04–1.74	0.026
No	695	12.1	11.1–13.1			Reference		
Brain metastasis				0.001				
Yes	228	9.2	7.6–10.9			1.18	0.98–1.43	0.079
No	561	12.6	11.4–13.8			Reference		
Bone metastasis				<0.0001				
Yes	325	10.4	9.3–11.6			1.24	1.04–1.48	0.016
No	464	13.0	11.8–14.1			Reference		
Pleura metastasis				<0.0001				
Yes	372	11.0	10.2–11.9			1.39	1.17–1.64	<0.001
No	417	13.2	11.3–15.1			Reference		
Adrenal metastasis				<0.001				
Yes	75	8.6	5.3–12.0			1.37	1.04–1.80	0.025
No	714	12.0	11.1–13.0			Reference		
Distant lymph node metastasis				0.481		-		
Yes	75	10.2	6.0–14.3					
No	714	11.7	10.8–12.6					
Pericardia metastasis				0.001				
Yes	10	1.8	1.2–2.5			2.39	1.16–4.92	0.018
No	779	11.7	10.8–12.6			Reference		
Peritoneum metastasis				0.102		-		
Yes	4	3.9	0.1–13.2					
No	785	11.7	10.8–12.6					
No. of metastatic sites				<0.0001		-		
0-1	348	14.6	12.3–16.9					
2-3	354	11.0	10.2–11.9					
4 or more	87	7.7	5.8–9.7					
EGFR-TKI				0.003		-		
Afatinib	312	14.7	13.0–16.3			Reference		
Erlotinib	259	10.8	9.7–11.9			1.17	0.97–1.42	0.102
Gefitinib	218	9.9	8.6–11.2			1.48	1.21–1.80	<0.001

Footnote: A series of univariate Cox proportional hazards models were performed to initially screen for potential factors associated with PFS. Those variables with p-values <0.05 in the univariate Cox analysis were further introduced into a multivariable Cox model. A two-sided p-value of <0.05 was considered statistically significant.

Abbreviations: CI, confidence interval; EGFR-TKI, epidermal growth factor receptor-tyrosine kinase inhibitor.

Multivariate analysis was performed to identify potential independent prognostic factors of PFS (Table 4). PS of 2–4, stage IV disease, liver, bone, pleural, adrenal, and pericardial metastasis, and EGFR-TKI treatment with gefitinib were independent unfavorable prognostic factors of PFS.

### Univariate and multivariate analysis of prognostic factors of overall survival of elderly patients

Univariate analyses were performed to explore the possible prognostic factors of OS of patients aged ≥65 years treated with EGFR-TKIs (Table 5). The OS of patients aged ≥85 years with a PS of 2–4, non-adenocarcinoma morphology, stage IV disease, and ≥4 metastatic sites was significantly worse. In addition, the OS of patients with liver, brain, bone, pleural, adrenal, and pericardial metastasis was significantly worse. EGFR-TKI treatment with gefitinib was also associated with significantly worse OS.

**Table 5:** Univariate and multivariate analysis of prognostic factors of overall survivals (OS) for elderly patients (age ≥ 65 years).

**Parameters**	**No**	**Univariate analysis**				**Multivariate analysis**		
		**Median** **(months)**	**95% CI**	***p-*Value**		**Hazard ratio**	**95% CI**	***p-*Value**
Age (years)				<0.0001				
65–74	383	23.0	20.3–25.6			Reference		
75-84	323	19.0	16.7–21.2			1.17	0.98–1.38	0.077
≥85	83	9.0	5.8–12.3			1.90	1.45–2.48	<0.0001
Sex				0.512		-		
Male	305	20.5	17.8–23.2					
Female	484	19.5	17.1–21.8					
Performance status				<0.0001				
0-1	588	24.1	21.8–26.5			Reference		
2–4	201	8.0	5.7–10.4			2.21	1.84–2.65	<0.0001
Smoking				0.092		-		
Yes	158	17.7	12.7–22.8					
No	603	20.3	18.0–22.5					
Unknown	28	16.7	0.9–32.4					
Tumor morphology				0.046				
Adenocarcinoma	771	20.2	18.3–22.1			Reference		
Non-adenocarcinoma	18	8.0	0.4–15.5			1.11	0.67–1.84	0.697
Mutation				0.187		-		
19del	344	22.0	18.8–25.3					
L858R	445	18.2	16.0–20.3					
Stage				<0.0001				
IIIB	67	45.1	31.1–59.0			Reference		
IV	722	18.8	17.0–20.5			1.80	1.25–2.59	0.002
Lung metastasis				0.059		-		
Yes	319	19.0	15.4–22.5					
No	470	20.7	18.2–23.3					
Liver metastasis				<0.0001				
Yes	94	11.5	8.8–14.1			1.43	1.12–1.82	0.004
No	695	20.9	19.2–22.7			Reference		
Brain metastasis				<0.0001				
Yes	228	14.2	11.4–16.9			1.22	1.02–1.47	0.030
No	561	21.8	19.5–24.2			Reference		
Bone metastasis				<0.0001				
Yes	325	14.7	12.3–17.2			1.43	1.20–1.69	<0.0001
No	464	23.5	20.6–26.5			Reference		
Pleura metastasis				<0.001				
Yes	372	17.2	14.9–19.6			1.34	1.13–1.57	<0.001
No	417	22.8	20.2–25.3			Reference		
Adrenal metastasis				<0.0001				
Yes	75	12.5	10.0–14.9			1.53	1.18–1.99	0.001
No	714	20.9	18.9–22.8			Reference		
Distant lymph node metastasis				0.406		-		
Yes	75	17.7	13.3–22.1					
No	714	20.3	18.2–22.3					
Pericardia metastasis				<0.001				
Yes	10	1.8	0.1–7.1			2.54	1.33–4.88	0.005
No	779	20.1	18.2–22.1			Reference		
Peritoneum metastasis				0.954		-		
Yes	4	3.9	0.1–20.6					
No	785	20.1	18.2–22.1					
No. of metastatic sites				<0.0001		-		
0-1	348	27.2	23.9–30.7					
2-3	354	17.2	14.8–9.5					
4 or more	87	7.8	4.4–11.2					
EGFR-TKI				0.026				
Afatinib	312	22.2	18.6–25.9			Reference		
Erlotinib	259	18.5	14.9–22.1			1.05	0.87–1.27	0.623
Gefitinib	218	17.7	13.7–21.7			1.27	1.05–1.55	0.016

Footnote: A series of univariate Cox proportional hazards models were performed to initially screen for potential factors associated with OS. Those variables with p-values <0.05 in the univariate Cox analysis were further introduced into a multivariable Cox model. A two-sided p-value of <0.05 was considered statistically significant.

Abbreviations: CI, confidence interval; EGFR-TKI, epidermal growth factor receptor-tyrosine kinase inhibitor.

Multivariate analysis was performed to identify the potential independent prognostic factors of OS (Table 5). Age ≥85 years, PS of 2–4, stage IV disease, liver, brain, bone, pleural, adrenal, and pericardial metastasis, and EGFR-TKI treatment with gefitinib were independent unfavorable prognostic factors of OS.

## DISCUSSION

This large retrospective study used real-world data to determine the effectiveness and safety of EGFR-TKIs for elderly patients with *EGFR*-mutated advanced NSCLC. Of 789 patients aged ≥65 years, 218 were treated with gefitinib, 259 with erlotinib, and 312 with afatinib. Younger patients were more frequently treated with afatinib than older patients (58.3% vs. 39.6%; *p* < 0.0001). However, afatinib remained the preferred TKI for older patients compared to erlotinib or gefitinib. Afatinib as a first-line treatment was more effective, with a median PFS of 14.7 months and OS of 22.2 months, than gefitinib (9.9 and 17.7 months, respectively) and erlotinib (10.8 and 18.5 months, respectively). However, patients treated with afatinib also experienced more grade ≥3 AEs than those treated with gefitinib or erlotinib. Furthermore, PS of 2–4, stage IV disease, liver, bone, pleural, adrenal, and pericardial metastasis, and EGFR-TKI treatment with gefitinib were identified as independent unfavorable prognostic factors of PFS, while age ≥ 85 years, PS of 2–4, stage IV disease, liver, brain, bone, pleural, adrenal, and pericardial metastasis, and EGFR-TKI treatment with gefitinib were independent unfavorable prognostic factors of OS.

In the LUX-Lung 3 study, PFS of patients with *EGFR*-mutated advanced lung adenocarcinoma was longer with afatinib than with doublet chemotherapy (11.1 vs. 6.9 months; HR = 0.58, 95% CI = 0.43–0.78, *p* = 0.001) [5]. In the LUX-Lung 7 study, PFS of patients with advanced NSCLC with common *EGFR* mutations was longer with afatinib than with gefitinib (11.0 vs. 10.9 months; HR = 0.73, 95% CI = 0.57–0.95, *p* = 0.017) [9]. However, it is important to note that the results of these studies may not necessarily apply to all populations, including elderly patients. In the LUX-Lung 7 study, the only randomized study comparing afatinib and first-generation EGFR-TKIs, the median age of both groups was 63 years and 44.5% of the patients were more than 65 years old. The benefit of afatinib for this subgroup was nonsignificant with an HR of 0.85 (95% CI = 0.59–1.22), which might have been due to the small number of cases [9]. In addition, the safety of TKIs for elderly patients was rarely discussed in LUX-LUNG 7 and retrospective studies.

Real-world evidence shows that the survival outcomes of patients with advanced NSCLC with common *EGFR* mutations [10], uncommon *EGFR* mutations [12, 14, 15], or poor PS [16, 17] were better with afatinib than with gefitinib or erlotinib. In a study of 2190 patients with common *EGFR* mutations, univariate analysis identified EGFR-TKI use as a prognostic factor (erlotinib or gefitinib vs. afatinib; *p* < 0.0001). Multivariate analysis confirmed EGFR-TKI use as an independent prognostic factor (erlotinib vs. afatinib: adjusted HR [AHR] = 1.274, 95% CI = 1.117–1.454, *p* < 0.001; gefitinib vs. afatinib: AHR = 1.461, 95% CI = 1.307–1.633, *p* < 0.0001) [10]. In a study of 230 patients with uncommon *EGFR* mutations, PFS and OS of patients receiving afatinib were better than those of patients receiving gefitinib or erlotinib (PFS: 6.4 vs. 5.9 months, *p* = 0.022; OS: 13.4 vs. 13.0 months, *p* = 0.008) [12]. Similarly, in an investigation of 517 patients with a PS ≥2 [16], PFS and OS of patients treated with 40 mg of afatinib were better than those of patients treated with gefitinib or erlotinib (PFS: 11.6 vs. 6.8 or 6.7 months, *p* = 0.009; OS: 16.2 vs. 10.0 or 9.6 months, *p* = 0.001), although this trend was nonsignificant in multivariate analyses. Dose adjustment was an independent prognostic factor of PFS and OS, regardless of the EGFR-TKI used [16].

While most patients with *EGFR*-mutated NSCLC initially respond well to EGFR-TKIs, the disease eventually progresses due to acquired resistance. A secondary *EGFR* mutation involving a substitution of threonine for methionine at position 790 (T790M) has been identified [18]. Osimertinib can overcome treatment resistance associated with this *EGFR* T790M mutation, with afatinib followed by osimertinib being an effective therapeutic strategy [11, 19–21].

Patients discontinuing EGFR-TKIs due to intolerable AEs should be switched to another EGFR-TKI [22]. In a retrospective study of 2190 patients treated with first-line EGFR-TKIs, 114 experienced intolerable AEs requiring discontinuation of EGFR-TKIs. Age >65 years, female sex, body weight, and body surface area were associated with intolerable AEs in patients treated with afatinib. PFS of patients receiving subsequent first-line EGFR-TKIs (median = 14.9 months, 95% CI = 11.0–18.8 months) was better than that of patients receiving chemotherapy (median = 7.0 months, 95% CI = 1.0–12.3 months) and patients without subsequent treatment (median = 0.9 months, 95% CI = 0.6–1.2 months). In addition, OS of patients receiving subsequent EGFR-TKIs (median = 31.3 months, 95% CI = 23.9–38.7 months) was better than that of patients receiving chemotherapy (median = 19.4 months, 95% CI = 18.5–20.3 months) and patients without subsequent treatment (median = 2.4 months, 95% CI = 1.3–3.5 months) [22].

This study has several limitations. Bias might have been introduced into the study due to its retrospective nature. There might also have been selection bias since the clinician chose the EGFR-TKI. In addition, the choice of sequential treatment could have affected survival outcomes.

In conclusion, this study demonstrated the effectiveness and safety of EGFR-TKIs for elderly patients with *EGFR*-mutated advanced NSCLC, a population that has often been underrepresented in clinical trials and real-world evidence. For elderly patients with *EGFR*-mutated advanced NSCLC, clinicians were more likely to prefer gefitinib or erlotinib to afatinib as a therapy, in contrast to the treatment regimen for younger patients. Nevertheless, afatinib still emerged as the primary choice for first-line treatment for older patients compared to other EGFR-TKIs, as it is more effective than gefitinib or erlotinib in elderly patients with *EGFR*-mutated advanced NSCLC.

## Supplementary Material

Supplementary Figure 1

Supplementary Table 1
