# Supplementary material for: Effectiveness and safety of afatinib, gefitinib, and erlotinib for treatment-naïve elderly patients with epidermal growth factor receptor-mutated advanced non-small-cell lung cancer: a multi-institute retrospective study
Source: Aging (Albany NY). 2024 Jan 8;16(1):550–67. doi: 10.18632/aging.205395 (PMC10817377; doi:10.18632/aging.205395)
Supplement: Supplementary Figure 1 [file aging-16-205395-s001.pdf]

SUPPLEMENTARY FIGURE

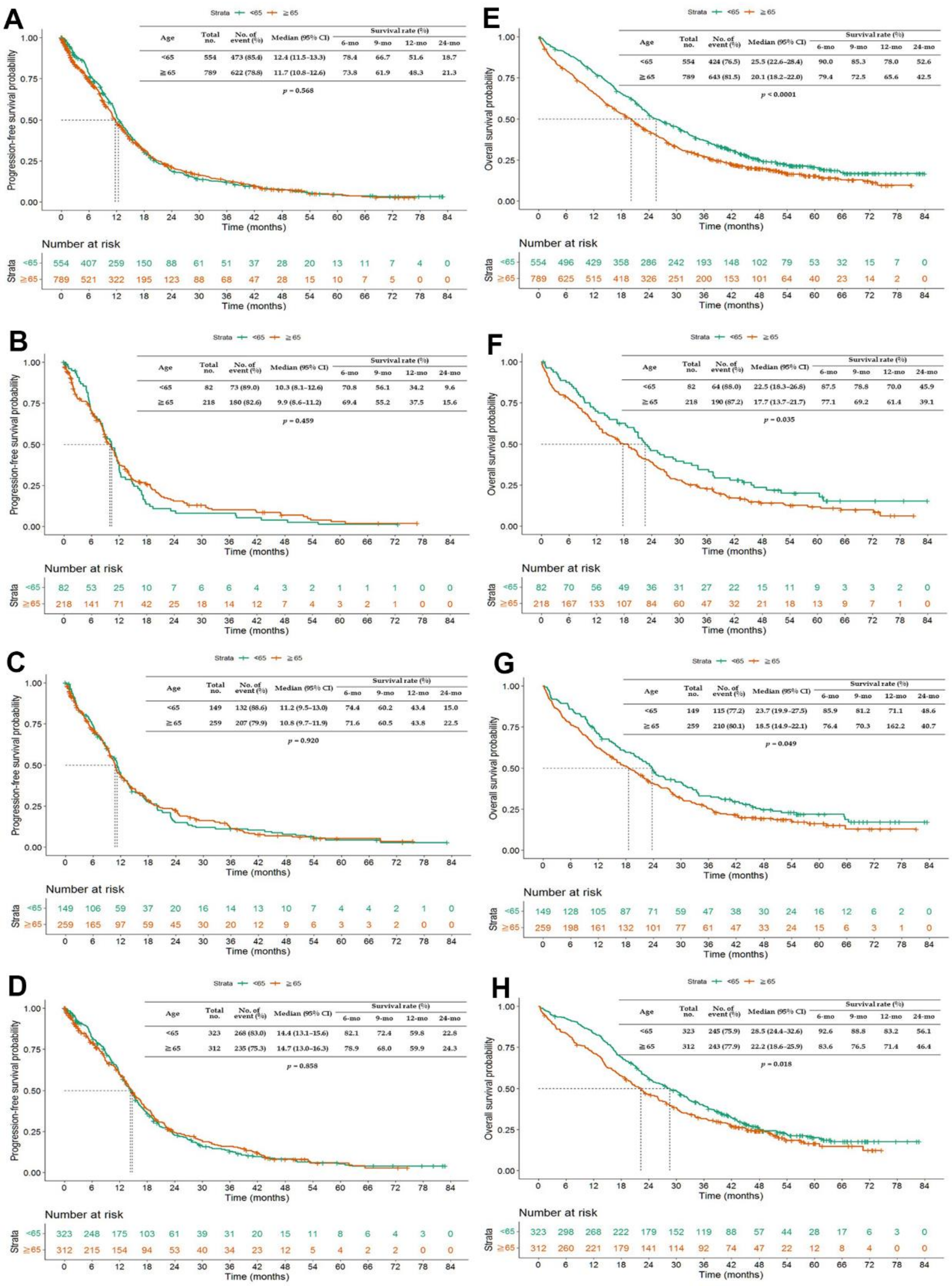

Supplementary Figure 1. Kaplan–Meier curves of progression-free survival (PFS) and overall survival (OS) of younger patients (age <65 years) and older patients (age ≥65 years) treated with epidermal growth factor receptor-tyrosine kinase inhibitors (EGFR-TKIs). (**A**) PFS for all EFGR-TKIs; (**B**) PFS for gefitinib; (**C**) PFS for erlotinib; (**D**) PFS for afatinib; (**E**) OS for all EGFR-TKIs; (**F**) OS for gefitinib; (**G**) OS for erlotinib; and (**H**) OS for afatinib. Abbreviation: CI, confidence interval.
