# Supplementary material for: Effectiveness and safety of afatinib, gefitinib, and erlotinib for treatment-naïve elderly patients with epidermal growth factor receptor-mutated advanced non-small-cell lung cancer: a multi-institute retrospective study
Source: Aging (Albany NY). 2024 Jan 8;16(1):550–67. doi: 10.18632/aging.205395 (PMC10817377; doi:10.18632/aging.205395)
Supplement: Supplementary Table 1 [file aging-16-205395-s002.pdf]

SUPPLEMENTARY TABLE

Supplementary Table 1. Patients' characteristics between younger patients (age < 65 years) and older patients (age ≥ 65 years).

Characteristics	Age (years)		*p*-Value
	<65 (N = 554)	≥65 (N =789)	
Sex			0.697
Male	220 (39.7%)	305 (38.7%)	
Female	334 (60.3%)	484 (61.3%)	
Performance status			<0.0001
0	128 (23.1%)	105 (13.3%)	
1	340 (61.4%)	483 (61.2%)	
2	58 (10.5%)	119 (15.1%)	
3	22 (4.0%)	50 (6.3%)	
4	6 (1.0%)	32 (4.1%)	
Smoking			0.015
No	400 (72.2%)	603 (76.5%)	
Yes	143 (25.8%)	158 (20.0%)	
Unknown	11 (2.0%)	28 (3.5%)	
Tumor morphology			0.273
Adenocarcinoma	546 (98.6%)	771 (97.7%)	
Non-adenocarcinoma	8 (1.4%)	18 (2.3%)	
Mutation			<0.0001
Exon 19 deletion	247 (44.6%)	445 (56.4%)	
L858R	307 (55.4%)	344 (43.6%)	
Stage			0.032
IIIB	30 (5.4%)	67 (8.5%)	
IV	524 (94.6%)	722 (91.5%)	
EGFR-TKI			<0.0001
Afatinib	323 (58.3%)	312 (39.6%)	
Erlotinib	149 (26.9%)	259 (32.8%)	
Gefitinib	82 (14.8%)	218 (27.6%)	
Liver metastasis			0.069
Yes	85 (15.3%)	94 (11.9%)	
No	469 (84.7%)	695 (88.1%)	
Brain metastasis			0.001
Yes	207 (37.4%)	228 (28.9%)	
No	347 (62.6%)	561 (71.1%)	
Lung metastasis			0.425
Yes	212 (38.3%)	319 (40.4%)	
No	342 (61.7%)	470 (59.6%)	
Bone metastasis			<0.0001
Yes	298 (53.8%)	325 (41.2%)	
No	256 (46.2%)	464 (58.8%)	
Pleura metastasis			0.002
Yes	214 (38.6%)	372 (47.1%)	
No	340 (61.4%)	417 (52.9%)	
Adrenal metastasis			0.765
Yes	50 (9.0%)	75 (9.5%)	

No	504 (91.0%)	714 (90.5%)	
Distant lymph node metastasis			0.044
Yes	72 (13.0%)	75 (9.5%)	
No	482 (87.0%)	714 (90.5%)	
Pericardia metastasis			0.423
Yes	10 (1.8%)	10 (1.3%)	
No	544 (98.2%)	779 (98.7%)	
Peritoneal metastasis			0.654
Yes	1 (0.2%)	4 (0.5%)	
No	553 (99.8%)	785 (99.5%)	

Footnote: Continuous variables were compared using a t-test or analysis of variance. Categorical variables were compared using a Chi-square or Fisher's exact test.
Abbreviations: EGFR-TKI, epidermal growth factor receptor-tyrosine kinase inhibitor.
